# Defining Cultural Care for Immigrant Women Through Leininger's Sunrise Model: A Qualitative Study

**DOI:** 10.1002/nop2.70491

**Published:** 2026-03-12

**Authors:** Hafize Dağ Tüzmen, Betül Bayrak, Yağmur Sürmeli Akbal

**Affiliations:** ^1^ Faculty of Health Sciences KTO Karatay University Konya Turkiye; ^2^ Toros University Mersin Turkiye

**Keywords:** Leininger's Sunrise model, migrant women, nursing, qualitative study

## Abstract

**Aim:**

In this study, we aimed to describe the cultural care given to immigrant women based on Leininger's Sunrise Model.

**Design:**

A qualitative descriptive study.

**Methods:**

In this study, the ethno‐nursing research method, a distinctive approach within the nursing discipline introduced by Leininger through the Sunrise Model, was employed. The study was conducted between June and October 2023. 28 participants were included in the study and coded as P1‐P28.

**Results:**

As a result of the analysis, the data were collected under 12 themes. These themes are ethnicity, worldview, kinship and social factors, cultural values/beliefs and lifestyle, religious/spiritual/philosophical factors, technological factors, economic factors, political and legal factors, educational factors, language and communication factors, emic and ethical care beliefs and practices, general and special nursing/midwifery care factors. In addition, each theme was further subdivided into subcodes. In addition, each theme was divided into subcodes.

**Conclusion:**

According to the study findings, immigrant women prioritise values such as privacy, respect and sincerity; they need family and social support; religious beliefs and spiritual practices positively affect their psychological well‐being. In this context, it is recommended that transcultural training programs specific to immigrant health be disseminated to healthcare professionals, especially nurses and primary care workers, in order to increase cultural adaptation, reduce language barriers and integrate spiritual care. These trainings should include topics such as cultural sensitivity, effective communication, recognition of spiritual needs, confidentiality and respectful care.

**Implications for the Profession and/or Patient Care:**

It is thought that the results of this study will be an important guide for our nurse and midwife colleagues and other health professionals, especially in understanding the cultural care needs of immigrant women in health services and developing solutions for these needs.

**Patient or Public Contribution:**

No patient or public contribution.

## 1 Introduction

Madeline Leininger, a pioneer of cross‐cultural nursing, emphasised as early as the 1950s that healthcare services are influenced by factors such as culture, language, belief systems, environment and gender (Hotun Şahin et al. 2009; Leininger and McFarland 2005). Leininger's Sunrise Model, developed by Madeline Leininger and recognised as the first theoretical model used in cross‐cultural nursing, is based on integrating the individual's cultural values and lifestyle into care and aims to provide a holistic approach to culturally diverse groups (M. M. Leininger 1991). This aspect of the model can be of great benefit in developing cultural sensitivity in multicultural societies and regions with a high number of immigrants, such as Türkiye.

Türkiye has taken in a significant immigrant population in recent years due to rising migration flows. According to the Turkish Statistical Institute's Population Statistics Portal, the number of women who came to our country from other countries between 2016 and 2022 is around two million. When the nationalities of these women are considered, it is seen that the immigrants come from different geographical regions such as Syria, Afghanistan, Iran, Iraq, Palestine, and African countries (Turkish Statistical Institute Population Statistics Portal 2023). These data indicate that immigrant women are likely to encounter language and culture when accessing the healthcare system.

The literature review emphasises that migrant women are disadvantaged in accessing health services for reasons such as social isolation, loss of status, language barriers and limited social support, and that these barriers lead to unintended pregnancies, sexually transmitted infections, unsafe abortions, delayed antenatal care, inadequate family planning and increased perinatal/maternal risks (WHO 2018; Tasa et al. 2021; Mamuk and Şahin 2021; Letona et al. 2023; Pérez‐Sánchez et al. 2024). In this context, the provision of culturally sensitive health services that are sensitive to cultural differences, especially by nurses specialising in women's health, and the application of Leininger's Sunrise Model in care delivery are expected to play a crucial role in reducing the inequalities faced by migrant women. Leininger's Sunrise Model: Although it is a model that enables the implementation of holistic care that takes cultural diversity into account, its complexity and time demands may create challenges in clinical practice (Narayanasamy 2003; Cai 2016). Given the limited number of theoretical models that can shape nursing practice in the context of cultural nursing in Türkiye, this model can serve as a valuable framework. Therefore, this study aimed to explore and define the cultural care needs of immigrant women living in a province in the Central Anatolia Region of Türkiye, guided by Leininger's Sunrise Model.

## 2 Materials and Methods

### 2.1 Research Design

In this study, the ethno‐nursing research method was used as a guiding approach. This method, developed by Leininger, is a qualitative way of collecting and analysing data that focuses on understanding care through people's cultural values and everyday experiences (Leininger and McFarland 2005). Emerging in the mid‐1960s, it has become an important foundation for transcultural nursing practice (M. Leininger 2002). By using this approach, we were able to explore participants' beliefs, experiences, and perceptions of care in a culturally sensitive way. The interview questions were shaped to reflect this perspective, allowing participants to express themselves within their own cultural context. In this way, the ethno‐nursing method informed both the data collection and analysis processes and helped ensure that the findings were meaningful within the participants' cultural frameworks.

### 2.2 Population and Sample

The study population consisted of migrant women of childbearing age (18–49 years) living in Konya province. Information about the participation criteria was shared through social media, and women who showed interest were invited to (KTO Karatay University) for face‐to‐face interviews. Data collection continued until saturation was achieved. A purposive sampling method was used to include participants who met the predetermined criteria (Creswell 2013). The study was reported in line with the Consolidated Criteria for Reporting Qualitative Research (COREQ). In‐depth interviews were conducted with women who were fluent in Turkish and who voluntarily agreed to take part in the study.

### 2.3 Data Collection

The study was conducted between June and October 2023. One‐on‐one interviews were used to collect qualitative data from migrant women. The interviews were conducted by the lead researcher, who was introduced as the primary researcher with qualitative research experience, although she was not previously known to the participants. Social media platforms such as Instagram, Facebook, and WhatsApp were used to invite women aged 18–49 who could communicate in Turkish to participate in the study. As the study was designed to ensure accurate data collection and interpretation through direct communication, immigrant women who were able to speak Turkish were included. The decision to include Turkish‐speaking participants reflects a methodological choice aimed at maintaining the reliability of the data, rather than a limitation related to the researchers' language proficiency. Data were collected by face‐to‐face interviews with women who responded to the social media invitation and met the inclusion criteria at the KTO Karatay University, Nursing and Midwifery Laboratory. Consent was obtained before the interview. Each interview lasted approximately one hour. All sessions were transcribed by the lead researcher and coded with the consent of the participants. Before conducting the main study, a pilot study was conducted with two participants to assess the clarity and comprehensibility of the data collection tools. No changes were made to the data collection tools after the pilot study. The data obtained from the pilot study were not included in the analysis.

### 2.4 Data Collection Tools

Data were collected using a personal information form and an interview guide developed by researchers.

#### 2.4.1 Personal Information Form

The personal information form, developed by the researchers based on a thorough literature review, included six questions addressing the sociodemographic and obstetric characteristics of the participants. These questions included age, educational background, income level, marital status, employment status, and family type.

#### 2.4.2 Interview Form

The second component of the data collection tool was an interview guide developed based on Leininger's framework for evaluating cultural care. This framework consists of 12 key categories (These categories include: an individual's worldview; ethnohistory; kinship and social factors; cultural values, beliefs, and lifeways; religious, spiritual, and philosophical perspectives; technological influences; economic conditions; language and communication patterns; political and legal structures; educational factors; ethical and emic caregiving beliefs and practices and general and specialised nursing care factors), each facilitating an in‐depth exploration of culture‐related factors. In order to obtain comprehensive information on the ethnic background of the participants, questions were asked about their cultural background and the meaning of their family and social environment to them. In addition, the impact of values and belief systems, and religious, spiritual, and philosophical factors on health and well‐being were examined. Their views on the benefits of technology for health were also assessed. In the economic dimension, the relationship between the concept of money and health and the continuity of life and the impact of economic status on health were investigated. The effects of political and legal factors on well‐being and the positive contributions of education level on health were also discussed from the perspective of the participants. In the context of language and communication factors, the languages used and understood by the participants were identified. Regarding ethnic and ethical care beliefs and practices, foods that are considered taboo or forbidden in the cultural context were questioned. Finally, within the context of factors related to general and specialised midwifery care, the meaning that participants attributed to the concept of care in their own culture was elaborated. In addition, both general and specialised nursing care considerations were incorporated into the evaluation criteria.

Leininger's framework is considered an essential tool for gaining a comprehensive understanding of individuals' life contexts, addressing the complexities of cultural diversity, and ensuring culturally congruent healthcare delivery (M. Leininger 2002).

### 2.5 Data Analysis

Sociodemographic characteristics were analysed using statistical software. Numerical data and percentages were employed to present the statistical findings. The qualitative data were analysed using the thematic analysis approach proposed by Braun and Clarke (2006), which was conducted in six distinct and systematic stages (Braun and Clarke 2006). In the initial stage, the textual data were thoroughly reviewed to extract the overall meaning and context. In the second stage, the data were systematically coded and organised into a coherent dataset. The third stage focused on identifying potential themes by grouping related codes and corresponding data. The researchers then evaluated whether the identified themes adequately captured the essence of the entire dataset, ensuring comprehensive representation. In the fifth stage, each theme was clearly defined and labelled to ensure clarity and consistency. In the final stage, representative quotations were carefully selected to illustrate and support each theme.

### 2.6 Trustworthiness

Within the thematic analysis framework, data are interpreted within their specific context, and the researcher's subjectivity is acknowledged as a fundamental component of the knowledge creation process (Braun and Clarke 2006). To enhance the study's reliability, the interview questions were carefully evaluated by two experts prior to data collection to ensure their relevance and alignment with the research objectives. Furthermore, to strengthen the trustworthiness of the findings, the criteria of credibility, transferability, dependability, and confirmability, as outlined by Lincoln and Guba (1985), were rigorously followed. The inclusion of direct quotations from several participants also contributed to the study's transparency and methodological rigour (Lincoln and Guba 1985).

### 2.7 Ethical Considerations

The KTO Karatay University Non‐Drug and Medical Device Research Ethics Committee granted ethical approval to the study with its decision dated 22 June 2023 under reference number 2023/014. Throughout the research process, the four fundamental principles of ethical conduct—respect for persons, beneficence, non‐maleficence, and justice—were upheld (Beauchamp and Childress 2013). Prior to data collection, participants were provided with written information outlining the purpose and procedures of the study. The lead researcher ensured that all participants were fully informed of their right to voluntary participation, including the right to withdraw from the study at any time without any negative consequences.

## 3 Results

The study adopts a qualitative research model aimed at identifying the cultural care needs of immigrant women through the application of Leininger's Sunrise Model. A total of 28 participants were included, each assigned a unique identifier (P1 to P28). Sociodemographic analysis of the participants revealed a mean age of 26.25 ± 6.80 years. Of the participants, 64.3% were single, 35.7% were married, 10.7% were literate, 25% had completed primary education, and 64.3% were high school graduates. Employment status indicated that 17.9% of participants were unemployed, while 82.1% were employed. Regarding income, 10.7% reported having an income lower than their expenses, 71.4% stated that their income was equal to their expenses, and 17.9% reported an income exceeding their expenses. Additionally, 35.7% of participants lived in nuclear families, while 64.3% had extended family structures.

**FIGURE 1:**
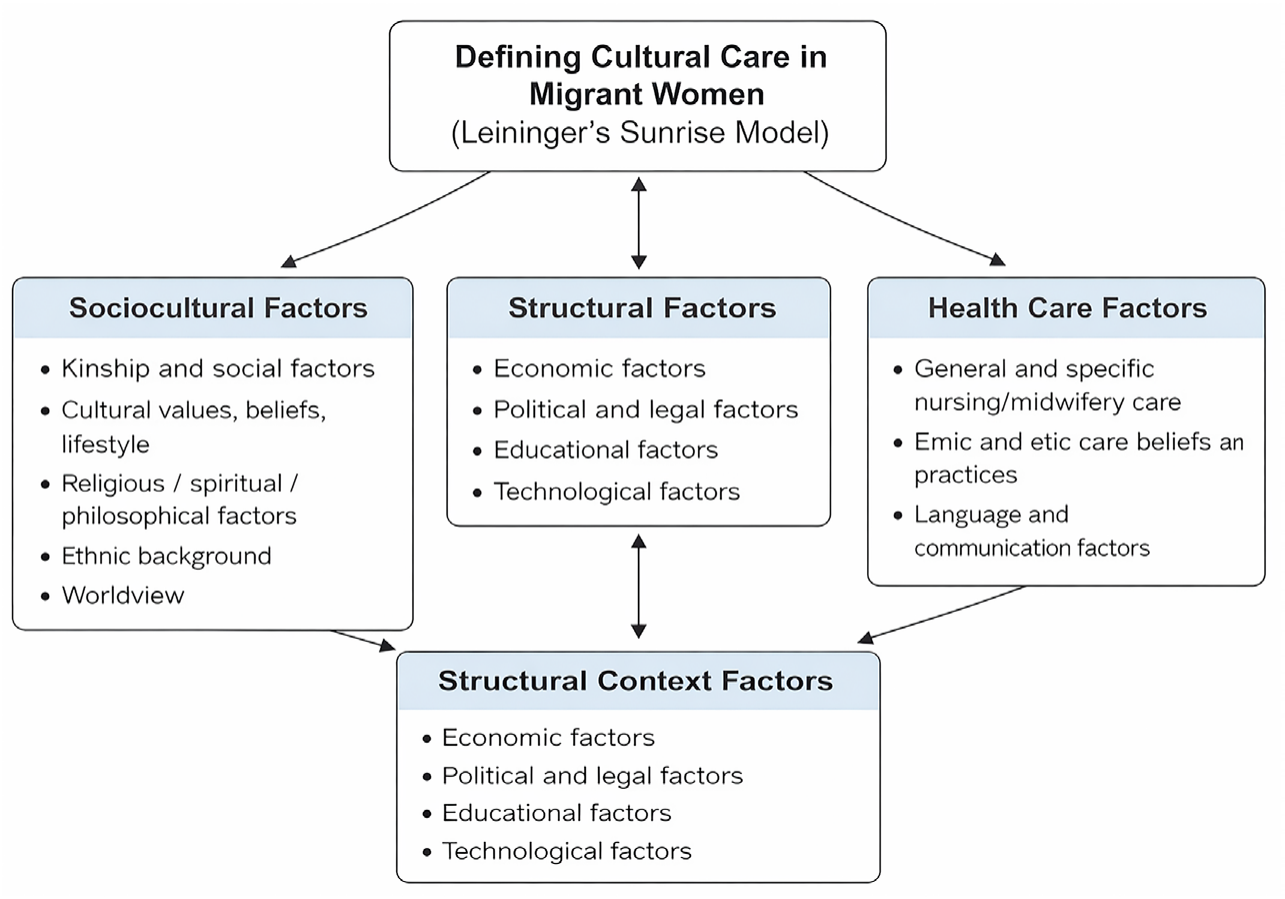
Theme display.

As shown in Figure 1, the study data were organised into 12 themes reflecting the different factors that shape cultural care. These themes are: ethnohistory; worldview; kinship and social factors; cultural values, beliefs, and lifeways; religious, spiritual, and philosophical perspectives; technological influences; economic conditions; political and legal structures; educational factors; language and communication patterns; ethical and emic caregiving beliefs and practices; and general and specialised nursing/midwifery care factors.

### 3.1 Theme 1: Ethnohistory

The first theme explored in this research pertains to language history, specifically focusing on the spoken languages of the participants. A category titled spoken language was established, consisting of 15 distinct codes. These codes represent the various languages spoken by the participants, including Turkish, Arabic, English, Russian, Uzbek, Kazakh, French, Bambara, Ethiopian, Oromo, Persian, Zan, Hausa, Songhai, and Ottoman Turkish. Some participant statements related to the subject are as follows:

I can speak Arabic, Turkish, and English, but I mostly prefer Arabic and Turkish. (P9)

Although most participants reported speaking more than one language, the languages most frequently used in daily life were Arabic and Turkish. This shows that language can act as both a bridge and a barrier to accessing health services. It also appears that language preferences are closely linked to the cultural context to which participants feel they belong.

### 3.2 Theme 2: Worldview

Twelve codes were identified within the second theme of the study, with focuses on participants' worldview. These codes reflect the diverse perspectives and experiences of the immigrant women, including a challenging environment, a place of endless exploration and adventure, rapidly developing and changing, striving for personal development, the brief span between life and death, experiences of injustice and racism, a holistic perspective of the world, material and spiritual negativity, complexity, a lack of sincerity and empathy, the beauty of life, and unique or special. Some participant statements related to the subject are as follows:

We live in a difficult world, both financially and spiritually. In our country, they sowed division between two groups, and we had to flee to avoid hurting our own people. I wish it hadn't happened this way. (P17)

The things I have witnessed have not been easy, especially due to the war. As the daughter of immigrants, I realized that I must work harder to improve myself, because if I don't do that, no one will offer me help. (P2)

The world is a brief journey that begins with birth and ends with death. Along the way, people face many trials, and it is a place where we can earn rewards for the afterlife. (P23)

Migrant women's worldviews are shaped by their exposure to traumatic experiences, war‐related events, and the process of adapting to a new society. While the majority of participants described the world as a place full of hardship, injustice, and constant struggle, others perceived life as a meaningful journey despite its challenges. Expressions such as ‘life is short but meaningful’ reflect efforts to reconstruct meaning in the face of the losses associated with migration. These findings indicate that the migration experience involves not only spatial relocation but cognitive and existential change.

### 3.3 Theme 3: Kinship and Social Factors

The third theme addressed in the scope of this research focuses on caregiving roles and family/friendship relationships, which were explored in two distinct categories. Within the first category of caregiving roles, thirteen codes were identified as follows: having individuals available to provide care, fulfilling familial responsibilities, requesting support when receiving care, the responsibility of providing care, support from the social environment, caregiving roles (e.g., mother, sister, mother‐in‐law, sister‐in‐law), receiving support from friends when in need of care, lack of responsibility for providing care, caregiving roles (e.g., spouse, friend, family), reluctance to receive care, not having individuals who can provide care, and the absence of family responsibility for caregiving. Some participant statements related to the subject are as follows:

Yes, they are very helpful; they do everything for me. (P4)

The individuals in my family are fulfilling their own duties and responsibilities. They do whatever they can given the situation. (P1)

For many immigrant women, care experiences are closely shaped by their relationships with family members and close social circles. Most participants described family support as central to the caregiving process, while friendships were often seen as a ‘second family’. This points to an effort to rebuild social networks after migration and highlights the ongoing need to feel a sense of belonging.

Within the category of family/friendship relationships, eight codes were identified. These codes include support from parents; the family as the primary source of support and security; friends providing social and psychological support (viewed as a second family); unconditional family support during both good and difficult times; love, trust, happiness, and peace; caring and supportive relationships (or lack thereof) with family and friends; the role of unity and togetherness in easing life; and the influence of personal relationships based on mutual trust and effort. Some participant statements related to this theme are as follows:

Family is the most important thing in my life. (P4)

Since I am a foreigner, I consider friendship to be like a second family. (P14)

A strong relationship is observed between this theme and ‘Cultural Values, Beliefs and Lifeways’. For example, how family responsibilities overlap with religious or cultural values shows that the perception of care is linked to providing not only physical support but also emotional security.

### 3.4 Theme 4: Cultural Values, Beliefs and Lifeways

The fourth theme examined in this research, cultural values, beliefs and lifeways, is divided into three categories: examples of care, cultural values, and beliefs. Within the examples of care category, six codes were identified. These include privacy, receiving attention when ill, respectful, sincere, and positive behaviour, helping those in need, maintaining a smiling face and patience, and avoiding unnecessary interventions while providing appropriate care. The participant statements related to the subject are as follows:

I would like my privacy to be respected. After my accident, I was really impressed by the nurses who took care of my privacy and always made sure I was covered. (P4)

I want my privacy to be prioritized, and I don't want to be examined by a male doctor. I can't think of any positive examples. (P16)

Within the cultural values category, eleven codes were identified. These codes reflect the diverse cultural perspectives and values that immigrant women bring with them to the healthcare setting. The identified codes are religious values, Arab culture, courtesy and respect, humanity, hospitality, aversion to lying, Afghan culture, African culture, kinship relations, Ottoman culture, and Uzbek culture. Some participant statements related to the subject are as follows:

I am a Muslim, and my cultural values are closely tied to my religion. I am committed to my religion, and respect, love, and cooperation within the family are the foundation of our values. (P4)

I come from an Arab culture. Thank God, I am a Muslim, but before that, I believe in humanity. (P14)

Within the beliefs category, four codes were identified. These codes reflect the diverse belief systems that immigrant women hold, which influence their perspectives on health, well‐being and caregiving practices. The identified codes are: Islamic belief, atheism, religious belief, and superstitious beliefs. The participant statements related to the subject are as follows:

There are certain beliefs in our culture. The main religion is faith, whether Muslim, Alevi, or Christian—it doesn't matter, as long as one is a believer. (P2)

The participants' cultural understanding of care is shaped around values such as confidentiality, respect, and sincerity. These values also reflect the family‐centred and belief‐driven care perceptions described under the theme of *Kinship and Social Factors* and extend into religious perspectives.

### 3.5 Theme 5: Religious, Spiritual and Philosophical Perspectives

The fifth theme explored in this research is divided into three categories as philosophical factors, spiritual factors, and religious factors. Nine codes were identified within the philosophical factors category as prioritising nursing/midwifery care, the importance of integrating religious care into nursing, spending time with loved ones and fostering communication, engaging in preferred activities, helping others feel better, applying traditional methods, watching TV series and motivational speeches, rejecting traditional practices, and maintaining a positive outlook on life. The first code within the philosophical factors category is prioritising nursing/midwifery care. Some participant statements related to the subject are as follows:

Both are important, but nursing/midwifery care comes first. If that's not enough, it is supported by religious care. (P13)

It makes me feel good when the person providing my treatment listens to me sincerely and takes care of me. (P23)

I believe it is always beneficial for a person to have faith. It also helps because we practice traditional methods with belief. (P17)

Within the spiritual factors category, four codes were identified as engaging in worship or prayer, deriving comfort from reading or listening to sacred texts, experiencing psychological and spiritual well‐being through religious practice, and exercising patience through faith. The participant statements related to the subject are as follows:

It makes me feel good to pray. (P13)

Yes, when we fully embrace our religion, we experience a state of psychological and spiritual health and well‐being. (P1)

Six codes were identified within the religious factors category, the third and final category of this theme. These codes include: the role of religion in emergency situations, the impact of religious practices, the priority of religious care, the perception that religion is not helpful or insufficient in emergency situations, the contribution of traditional/religious practices to well‐being, and the partial effectiveness of religious practices. Some participant statements related to the subject are as follows:

Absolutely, I believe that our religion provides a way of life. (P28)

Yes, during death, prayers are recited for the individual's soul as a religious practice to ease the pain of departure, and the declaration of martyrdom is recited. In times of emergency or illness, we believe it comes from Allah as a test, which helps us remain patient and calm. We find comfort and strength in religion in these situations. (P23)

Yes, it helps, but religious practices should take priority. (P10)

Participants described religious and spiritual beliefs as coping mechanisms and complementary elements in the caregiving process. These beliefs reinforce the value‐based care expectations previously described under *Cultural Values* and *Kinship and Social Factors*.

### 3.6 Theme 6: Technological Influences

Eight codes were identified within the technological influences theme, reflecting the role and impact of technology in the healthcare experiences of immigrant women. These codes are as follows: the importance of receiving hospital services, maintaining health through technology, the positive and negative impacts of technology, the role of advanced technology in improving healthcare quality, its significance in the early diagnosis and treatment of diseases, the partial effectiveness of technology in health development, the contribution of technology to knowledge and awareness, and the importance of technology in disease detection. Some participant statements related to the subject are as follows:

It is very important because the people working in the hospital know the best practices for our health better than we do and help us with that. (P4)

People can learn about diseases through technology, which also makes treatment more accessible and improves communication. The production of medical tools and devices, which is life‐saving, is made possible through technology. (P23)

The majority of participants evaluated technology as an element that facilitates access to the health system and increases health literacy. It is understood that mobile applications, diagnostic devices and digital information sources are positively received by immigrant women. This situation shows that technological tools serve as a bridge in the adaptation process to health services. This theme is closely linked to economic and educational factors that shape access to technological tools.

### 3.7 Theme 7: Economic Conditions

The theme is divided into three categories as health and money, factors supporting family livelihood, and monetary expression. Six codes were identified within the health and money category to reflect the complex relationship between financial factors and health outcomes of immigrant women. These codes include: the influence of money on health, failure to accumulate resources for health, inability to earn a living due to illness, financial difficulties related to health, saving for health, and the ineffectiveness of money in improving health. Some participant statements related to the subject are as follows:

Yes, we may need to undergo some treatments in private hospitals, and this is not possible without money. (P3)

No, I'm not saving money for my health. I suppose that's why I sometimes have difficulty finding money. (P1)

Five codes were identified within the factors supporting family livelihood category as family livelihood, parents, spouse/child, siblings, and personal factors. Some participant statements related to the subject are as follows:

We provide for the family; there are people in the family who can work. (P5)

Six codes were identified within the monetary expression category including money as an important factor for sustaining and easing life, money as everything, money as power, comfort, and freedom, money as a symbol of reputation, self‐confidence, and success, money as unimportant, and money as a basic necessity. Some participant statements related to the subject are as follows:

It's an important factor for us to move on with our lives, but money is not everything at the end of the day. (P4)

It is the means of acquiring almost everything. (P18)

Economic conditions emerged as a major barrier to healthcare access for immigrant women. Participants reported limited financial resources, which negatively affected both access to and continuity of care. Money was associated not only with health but also with power, freedom, and social status. Differences in participants' views suggest that perceptions of economic value are shaped by both cultural and individual factors.

### 3.8 Theme 8: Political and Legal Structures

The eighth theme of the study addressed political and legal structures, within which two distinct codes were identified: the impact of political factors and the perceived inefficacy of political structures. Some participant statements related to the subject are as follows:

Health policy is behind a lot of the global health problems we see today. There are several issues we are dealing with, like outbreaks of diseases that vaccines could prevent, such as measles and diphtheria. There's also the rise of drug‐resistant infections, more people becoming obese and not being active, and the impact of pollution and climate change on public health. (P21)

I don't think it has an impact. (P17)

This theme shows how immigrant women view the healthcare system from a structural and political perspective. Participants felt that health policies can contribute to inequalities and connected issues such as preventable diseases, poverty, and environmental problems to wider systemic shortcomings. Some described a sense of distance between policymakers and their own lived realities, expressing feelings of alienation and underrepresentation. This theme is also linked to (Language and Communication Patterns) and (Obstacles in Care), as policy‐related gaps directly affect both the quality and accessibility of health services.

### 3.9 Theme 9: Educational Factors

Under the educational factors theme, five codes were identified, reflecting the impact of education on health management and disease prevention. These codes are as follows: the positive influence of health management in educated individuals, the effectiveness of education in promoting health and preventing disease, the insufficiency of education in ensuring good health, partial management of health in educated individuals, and the partial importance of education in disease prevention.

The first code, the positive influence of health management on educated individuals, was particularly highlighted by participants, who noted that an individual with an education is better equipped to make informed decisions about their health. Some participant statements related to the subject are as follows:

Of course, they can take care of themselves because they know everything. Sometimes, just knowing how to read is enough. (P16)

Yes, when we feel unwell, we immediately look into it and treat it accordingly. We can also take preventive measures because we've learned about harmful things and stopped using them. (P23)

I don't believe that every educated person can manage their health positively, because I associate this with awareness. It's more about being conscious of one's own health and care, rather than just having an education. (P7)

The statements of immigrant women show that education plays an important role in accessing health information, developing protective behaviours, and making informed decisions. Participants noted that educated individuals are often able to recognise health risks earlier and take appropriate precautions. However, some also pointed out that education alone is not enough, and that personal health awareness is a key factor. These views suggest that education and awareness may not always develop at the same level. This theme is closely related to Technological Influences and Health Literacy, as education can make it easier to use health technologies and engage with the healthcare system.

### 3.10 Theme 10: Language and Communication Patterns

Within the language and communication pattern's theme, a category focusing on experienced challenges was developed, resulting in the identification of nine codes. These codes reflect various communication barriers and challenges immigrant women face, particularly related to their experiences with language, racism, and healthcare interactions. The nine codes are as follows: communication difficulties, racism/discrimination, absence of problems, language‐related issues, impact of cross‐country transition on lifestyle, adaptation challenges associated with relocation, prejudice, issues related to nurse workload, and the desire to avoid experiencing racism. Some participant statements related to the subject are as follows:

Unfortunately, there are people who display undesirable traits like racism, extremism, and prejudice based on race or color, and these issues often lead to misunderstandings. This is particularly common in communication with nurses because of language differences. (P11)

Yes, I've experienced this a lot. For example, sometimes when I go to the hospital and they see my place of birth in the system, the way they communicate with me changes. I often feel prejudice and racism. (P24)

Language and communication represent critical thresholds in immigrant women's access to healthcare. Reported experiences of misunderstanding, exclusion, and discrimination illustrate how communication problems directly influence care experiences, which are further detailed under the theme of *General and Specialised Nursing/Midwifery Care Factors*.

### 3.11 Theme 11: Ethical and Emic Caregiving Beliefs and Practices

Within the eleventh theme of beliefs and practices related to ethical and emic care, four categories were identified, focusing on the cultural, religious, and traditional health practices of immigrant women. The following eight codes emerged within the category of traditional/religious belief practices: avoidance of religiously prohibited foods, abstinence from alcohol and drugs, use of traditional treatments for wound healing and pain relief, the psychological effects of traditional treatments, the use of herbal teas and aromatherapy for healing, rejection of traditional treatment methods, cupping therapy, and treatments for hair loss. Some participant statements related to the subject are as follows:

According to Islam, we avoid intoxicating drinks and substances, as well as pork, blood, and dead animals. (P2)

If cupping is considered a traditional method, it helped me a lot. That's how my rheumatism was relieved. (P24)

Seven codes were identified within the category of health factors: the positive impact of a healthy lifestyle on well‐being and freedom; attention to meeting health and bodily needs; support from social interactions; psychological and physical well‐being; challenges associated with illness, fatigue, and general malaise due to illness; and the impact of increased responsibilities on overall well‐being. The participant statements related to the subject are as follows:

Sports activities, proper nutrition, and regular check‐ups 2–3 times a year are all important for my well‐being. (P21)

Four codes were identified within the category of professional/traditional treatment methods, including: the complementary nature of professional and traditional treatment methods, preference for professional health services, the safety and expertise associated with professional treatment methods, and the role of traditional treatment as an adjunct to professional care. The participant statements related to the subject are as follows:

I think, in general, both traditional and professional treatments support each other. It would be better if those involved in traditional treatments also developed professionally. (P1)

Professionals are experts in this field and have a deeper understanding of it. Those practicing traditional treatments, on the other hand, can only be helped by professionals. (P4)

Seven codes were identified within the category of professional nursing/midwifery behaviours including clear and effective communication, provision of education related to disease and treatment during care, demonstration of positive behaviour and empathy, avoidance of discriminatory, racist, or prejudicial behaviour, respect for patients and responsible conduct, maintaining calm and understanding, and displaying a warm, sincere demeanour. The participant statements related to the subject are as follows:

There should be no communication problems, and health professionals should not treat us, as immigrants, with racism or prejudice. (P1)

The beliefs and practices of immigrant women regarding healthcare are shaped by cultural, religious, and traditional knowledge systems. These practices influence expectations from professionals and care delivery, as further reflected in the theme of *General and Specialised Nursing/Midwifery Care Factor*.

### 3.12 Theme 12: General and Specialised Nursing/Midwifery Care Factors

Two categories were identified within the theme of general and specialised nursing/midwifery care. These categories include nursing/midwifery care factors and obstacles encountered in care delivery. Sixteen codes emerged within the nursing/midwifery care factors category, including: continuity of health maintenance through nursing care, care as a means of assistance, easing life and meeting needs, a preference for midwife/nurse care, professionalism and ethical standards, a warm and understanding demeanour, kindness and respect, reluctance to receive care from nurses/midwives, attention and responsiveness to care, peer support fostering trust, addressing informational gaps, absence of communication barriers, privacy and confidentiality, supportive care, patience and calmness, empathy, and the role of care in maintaining health. Some participant statements related to nursing/midwifery care factors include:

They should be kind and respectful. They should help and encourage me to participate in decisions about my care. They should respect my dignity, privacy, and rights. (P5)

I would feel embarrassed to ask for help. It can be difficult for me to have a conversation, especially about women's health. So, even if I wanted support, I might not be able to express it. (P2)

Nine codes were identified within the category of obstacles encountered in care delivery, including language and communication barriers, male nurse care, cultural differences, barriers to empathy, financial constraints, racism, challenges related to family planning, disrespect and irresponsibility, and feelings of alienation or lack of safety. Some participant statements related to the subject are as follows:

Language barriers and lack of empathy are obstacles to care. In our culture, we only work with female doctors, nurses, and midwives, and this can have an impact. (P16)

Of course, it does. Personal factors, legal and ethical issues, as well as cultural and social differences, all play a role. For example, early marriage is normal in our culture, but it is not accepted in Türkiye. (P22)

Immigrant women's expectations from healthcare extend beyond technical skills to encompass ethical conduct, quality communication, and cultural sensitivity. In women's health, gender preference, privacy protection, and empathetic care are crucial. Barriers in care affect not only physical but also cultural and emotional dimensions. Reluctance to receive care from male nurses, language barriers, and healthcare providers' prejudiced attitudes contribute to disengagement from the health system. This theme intersects with multiple factors including language, beliefs, economic status, and education. Inclusive and culturally sensitive care is essential to support the physical and mental health of immigrant women.

## 4 Discussion

This study explored the cultural care needs of immigrant women through the lens of Leininger's Sunrise Model and drew attention to the importance of culturally congruent care in health services. The findings suggest that immigrant women are more able to benefit from healthcare when care is delivered in a way that respects their cultural background. Participants often shared that when their beliefs, traditions, and language were taken into account, they felt more trust towards healthcare professionals and found it easier to adapt to the system. These observations echo Leininger's emphasis on integrating cultural values and belief systems into care.

Our research findings indicate that language barriers significantly hinder access to healthcare services. Participants reported that difficulties in communicating with healthcare professionals reduced their ability to benefit from these services. This is consistent with Bhugra et al.'s (2021) findings on the crucial role of language and culture in the integration of immigrants into new societies. Moreover, participants described not only efforts to cope with the challenges of migration but also attempts at personal growth and adaptation to a new community. Supporting this, Şahin's study suggests that migration can reshape people's worldviews and social roles, and that women in particular may experience significant personal and social changes during this process (Sahin 2020; Uzun 2021). In line with this, our study highlights the need for care that is both culturally sensitive and holistic to improve immigrant women's access to healthcare, and points to Leininger's Sunrise Model as a useful guide in understanding these needs.

According to our findings, immigrant women's emphasis on values such as privacy, respect, and sincerity closely relates to the cultural values and lifeways described in Leininger's Sunrise Model. Their understanding of privacy is strongly shaped by religious beliefs and family structures, which calls for clear boundaries to be maintained during the care process. This shows the empirical correspondence between the religious‐philosophical and social structure areas of the model. Respect shapes not only the individual's relationship with the caregiver, but also their expectations regarding the service process. It was observed that sincerity was seen as a ‘trust indicator’ in social interactions, and thus the participants felt more comfortable expressing themselves. This confirms the culturally dependent diversity of relational and individual behaviour patterns in Leininger's model. The findings of Scoppa and Stranges (2019) show that cultural norms play a decisive role in women's participation in business life and indicate that immigrant women's transfer of their perceptions of privacy and social roles to health services should be understood not only at the individual level but also within a broader cultural‐structural context (Scoppa and Stranges 2019). In this regard, our findings suggest that Leininger's model offers a useful framework for understanding cultural structures, but it may be less effective in capturing individual differences and personal changes over time. As noted by Liw et al. (2022), social interactions that align with cultural values can also support psychological well‐being. This shows that immigrant women's efforts to preserve values such as closeness are important not only for social integration but also for emotional resilience. While Leininger's model helps integrate cultural values into care, it is less clear how psychological dimensions are addressed, which points to some practical limits of the model. Overall, the results support Leininger's model for its strength in understanding cultural diversity but also highlight its shortcomings in accounting for individual and psychological factors. This suggests that approaches to cultural care should be more comprehensive, flexible, and include psychosocial dimensions.

According to our study findings, family and social support are critical in the lives of immigrant women. Participants stated that caregiving responsibilities are mostly met by family and close circle. This result is consistent with Alhasanat‐Khalil et al. (2018) who showed that lack of social support increases postpartum depression in immigrant women of Arab origin. However, some participants preferred public support mechanisms over religious or spiritual support; this situation is different from Alhasanat‐Khalil's study. Like Kara et al. (2023), our findings also revealed that family ties are decisive in the adaptation process. While Neufeld et al. (2002) model emphasises family‐centred support networks, we observed that some participants turned to neighbourhood relations and ties with nurses instead of social support because they were far from their families. In addition, Zanjari et al. (2022) and Gallardo‐Peralta et al. (2018) support the positive effect of social support on the quality of life and psychological well‐being of immigrant women. In our study, social support was linked not only to psychological well‐being but also to how immigrant women access and use healthcare services. These findings align with existing literature while offering new insights into how different sources of social support vary across cultural contexts. Additionally, religious beliefs and spiritual practices were found to strengthen the psychological well‐being of some immigrant women. This is consistent with the findings of Callender et al. (2022) and Ozeto and Allan (2021), who highlight the role of spirituality in coping with stress. However, this effect was not uniform among all participants; some viewed their faith as a source of inner strength, while others felt that it sometimes led them to distance themselves from modern healthcare. This highlights a potential tension between the religious‐philosophical component and the healthcare systems described in Leininger's Sunrise Model. Exploring these tensions suggests that culturally competent care should not only respect cultural values but also critically consider how these values influence access to and use of health services. Economic challenges were experienced differently among participants; some benefited from free healthcare services, whereas others faced significant barriers due to lack of insurance and limited financial resources. As Kusunose and Rignall (2018) emphasise, the economic dimensions of migration fundamentally shape how individuals access and use health services. An important insight from our findings is that even when women share similar economic conditions, they cope in different ways. Some rely on traditional support networks, while others seek to navigate the formal healthcare system. In this context, our study shows not only how cultural and spiritual values can positively influence health, but also how these values may sometimes create tension with access to services. Recognising these contrasts helps us better understand both the strengths and the limitations of Leininger's model in practice.

According to our study findings, a limited number of participants stated that lack of access to technological tools made it difficult to receive health services. This statement was especially evident in immigrant women who had difficulty using mobile health applications and digital appointment systems. These findings are consistent with Macharia and Maroa's (2014) need to increase the accessibility of Homeless Management Information System for immigrant groups (Macharia and Maroa 2014). However, data on how the more widespread and effective use of technology affects immigrant women's access to health services are limited in this study. This suggests that the theme plays a secondary role in immigrant women's experiences.

The findings of this study show that Leininger's Sunrise Model provides a helpful framework for understanding the healthcare experiences of immigrant women. Issues such as language barriers, privacy, expectations of respect and closeness, religious beliefs, and social roles clearly relate to the areas described in the model. It helps explain how cultural patterns influence care practices. At the same time, aspects like individual differences, psychological resilience, and the role of digital health tools seem to fall outside the model's main focus. These observations suggest that approaches to cultural care may need to be expanded to better include personal and psychosocial dimensions.

## 5 Conclusion and Recommendations

This study provides valuable guidance for understanding and addressing the cultural care needs of immigrant women in healthcare settings. The findings highlight the necessity of intercultural training for healthcare professionals to reduce language barriers, integrate spiritual care, and enhance cultural competence. Nurses, in particular, can offer more sensitive and inclusive care based on this model. Nurse educators can strengthen competencies by incorporating cultural care approaches into their curricula. Policymakers can support equitable healthcare access for immigrants through appropriate regulations and training policies. Future research could test the model's applicability across different cultures through comparative studies and explore ways to improve culturally sensitive care via technology‐based interventions.

## 6 Strengths and Limitations of Research

The use of Leininger's Sunrise Model in this research has facilitated a detailed understanding of the cultural expectations and needs of immigrant women in accessing healthcare services. The study thoroughly analysed the effects of language barriers, economic limitations, and cultural differences. However, since it was limited to immigrant women residing in a specific region, the generalizability of the findings is limited. This is because only immigrant women who can speak Turkish were included in this study. This situation limits the representation of the immigrant population by excluding the more vulnerable, newly arrived, or non‐Turkish speaking segments. This limitation limits the generalizability of the findings of the study to all immigrant women. Turkish speaking proficiency was determined as one of the basic criteria in participant selection. This is because the researchers do not speak common immigrant languages such as Arabic and Persian, and the participants do not know a universal language such as English. Although translator support was considered, this option was abandoned because it was anticipated that a translator who is not an expert in the field of health would be inadequate in conveying the meaning and this situation would negatively affect the data quality. In future studies, more inclusive sample groups can be created by targeting immigrant groups who speak a specific language with the support of researchers who know that language or health‐trained interpreters. In addition, intervention studies can be conducted to test intercultural education models and research can be conducted for quantitative validation of identified qualitative themes. In this way, it will be possible to examine the care experiences of more vulnerable immigrant groups in depth.

This study is one of the few qualitative investigations that comprehensively explore the cultural care experiences of immigrant women in Türkiye through the lens of Leininger's Sunrise Model. The adoption of an ethno‐nursing methodology enabled a profound understanding of the participants' cultural values, beliefs, and care expectations. A notable strength of the study is the systematic application of thematic analysis, complemented by direct participant quotations, which enhances both the reliability and transparency of the findings. In addition, by incorporating multidimensional sociocultural factors including language, family structure, religion, education, economic status, and technology, the study holistically captures the complex challenges immigrant women face in accessing culturally appropriate health services. The study also contributes to the validation and applicability of Leininger's model in a non‐Western context and enriches the global body of intercultural nursing literature.

## Author Contributions

**Hafize Dağ Tüzmen:** conceptualization, data collection, formal analysis, methodology, original draft writing, editing writing, review writing, references; **Betül Bayrak:** conceptualization, data collection, references; **Yağmur Sürmeli Akbal:** formal analysis, review writing, original draft writing, editing writing, references.

## Funding

This study was supported by KTO Karatay University BAP Commission (Project no: 17112436) and TUBITAK 2209 project.

## Conflicts of Interest

The authors declare no conflicts of interest.

## Data Availability

The data that support the findings of this study are available on request from the corresponding author. The data are not publicly available due to privacy or ethical restrictions.
